# Aspartic Proteases and Major Cell Wall Components in *Candida albicans* Trigger the Release of Neutrophil Extracellular Traps

**DOI:** 10.3389/fcimb.2017.00414

**Published:** 2017-09-21

**Authors:** Marcin Zawrotniak, Oliwia Bochenska, Justyna Karkowska-Kuleta, Karolina Seweryn-Ozog, Wataru Aoki, Mitsuyoshi Ueda, Andrzej Kozik, Maria Rapala-Kozik

**Affiliations:** ^1^Department of Comparative Biochemistry and Bioanalytics, Faculty of Biochemistry, Biophysics and Biotechnology, Jagiellonian University Krakow, Poland; ^2^Department of Analytical Biochemistry, Faculty of Biochemistry, Biophysics and Biotechnology, Jagiellonian University Krakow, Poland; ^3^Division of Applied Life Sciences, Graduate School of Agriculture, Kyoto University Kyoto, Japan

**Keywords:** neutrophil extracellular traps (NETs), *Candida albicans*, host defense, secreted aspartic proteases, mannans, glucans, fungal cell wall proteins

## Abstract

Neutrophils use different mechanisms to cope with pathogens that invade the host organism. The most intriguing of these responses is a release of neutrophil extracellular traps (NETs) composed of decondensed chromatin and granular proteins with antimicrobial activity. An important potential target of NETs is *Candida albicans*—an opportunistic fungal pathogen that employs morphological and phenotype switches and biofilm formation during contact with neutrophils, accompanied by changes in epitope exposition that mask the pathogen from host recognition. These processes differ depending on infection conditions and are thus influenced by the surrounding environment. In the current study, we compared the NET release by neutrophils upon contact with purified main candidal cell surface components. We show here for the first time that in addition to the main cell wall-building polysaccharides (mannans and β-glucans), secreted aspartic proteases (Saps) trigger NETs with variable intensities. The most efficient NET-releasing response is with Sap4 and Sap6, which are known to be secreted by fungal hyphae. This involves mixed, ROS-dependent and ROS-independent signaling pathways, mainly through interactions with the CD11b receptor. In comparison, upon contact with the cell wall-bound Sap9 and Sap10, neutrophils responded via a ROS-dependent mechanism using CD16 and CD18 receptors for protease recognition. In addition to the Saps tested, the actuation of selected mediating kinases (Src, Syk, PI3K, and ERK) was also investigated. β-Glucans were found to trigger a ROS-dependent process of NET production with engagement of Dectin-1 as well as CD11b and CD18 receptors. Mannans were observed to be recognized by TLRs, CD14, and Dectin-1 receptors and triggered NET release mainly via a ROS-independent pathway. Our results thus strongly suggest that neutrophils activate NET production in response to different candidal components that are presented locally at low concentrations at the initial stages of infection. However, NET release seemed to be blocked by increasing numbers of fungal cells.

## Introduction

*Candida albicans* is a common opportunistic fungal pathogen in humans that causes frequent superficial mucosal infections and, particularly in immunocompromised individuals, systemic infections (candidiasis) that can lead to sepsis and consequently death (Lichtenstern et al., 2015; Lagunes and Rello, 2016). The ability of *C. albicans* to infect different host niches is a consequence of its incredible adaptability to variable surrounding environments through morphogenetic and phenotypic switching from non-invasive yeast-like cells to a more virulent filamentous hyphal form (Calderone and Fonzi, 2001). This adaptability is also based on the ability of *C. albicans* to form biofilms on artificial and natural surfaces within the host organism. In all of these processes, *C. albicans* undergoes a continuous remodeling of its cell wall architecture via the activation of a wide range of virulence factors. The principal compounds exposed on the fungal cell surface, such as mannans and β-glucans, represent the main epitopes through which human host immune receptors respond to fungal infections (Chaffin et al., 1998; Collette and Lorenz, 2011). The other important group of surface compounds are proteins such as adhesins in the agglutinin-like sequence (Als) protein family which have a broad binding specificity for many human proteins (Liu and Filler, 2011; Karkowska-Kuleta and Kozik, 2015). In addition are the so-called “moonlighting proteins,” which are cytosolic proteins exposed on the fungal surface but whose function at this location remains unknown (Karkowska-Kuleta and Kozik, 2014).

Another group of candidal virulence factors includes a large family of secreted aspartic proteases (Saps) that not only facilitate the availability of nutrients for fungal growth (Mayer et al., 2013; Silva et al., 2014) but can also inactivate complement components (Gropp et al., 2009) and host antifungal peptides such as histatin or cathelicidin LL-37 (Rapala-Kozik et al., 2015; Bochenska et al., 2016), and cause the release of proinflammatory bradykinin-related peptides from kininogens (Rapala-Kozik et al., 2010; Kozik et al., 2015). Moreover, Saps are involved in the promotion of fungal cell adhesion to epithelial cells and tissues (Ibrahim et al., 1998). Saps also enable the escape and survival of fungal cells (Borg-von Zepelin et al., 1998) following an interaction with phagocytes and can serve as productive chemoattractants (Ran et al., 2013).

At the place of infection, *C. albicans* is detected by different immune cells, particularly by neutrophils (Netea et al., 2015). Neutrophils can kill microbes through phagocytosis, extracellularly through the release of antimicrobial factors via a degranulation process, or through the excretion of neutrophil extracellular traps (NETs) (Brinkmann et al., 2004). NETs are web-like structures that very effectively prevent pathogen spreading within the host and thus the further development of infections. NETs are composed of decondensed chromatin that is adorned with granular proteins such as elastase, myeloperoxidase (MPO), cathepsin G, and protease3, or with antibacterial peptides such as cathelicidin LL-37 (Brinkmann et al., 2004; Urban et al., 2009) that successfully combine to kill invading microbes.

NET formation, known as netosis, can be induced by bacteria, fungi, viruses, and parasites, as well as by activated platelets and some specific compounds such as cytokines, antibodies, and certain chemical substances. Netosis can also result from trauma (Brinkmann and Zychlinsky, 2012; Branzk and Papayannopoulos, 2013). The molecular mechanisms underlying netosis are still poorly understood but two main pathways have been described: (i) a classical mechanism that depends on the production of reactive oxygen species (ROS), with NADPH oxidase as the necessary signal mediator, and (ii) a rapid and ROS-independent mechanism (Rochael et al., 2015). The type of netosis pathway that is activated in different situations depends on the triggering factor and the receptors involved. The receptors involved in NET induction include the Toll-like receptors (e.g., TLR2, TLR4, CD14), C-lectin family (Dectin-1), complement receptors (CD11b/CD18; Mac-1), Fc-receptors (FcγRIIIb), and others (Yipp et al., 2012; Mohanty et al., 2015; Aleman et al., 2016). Moreover, most of these molecules can also function as co-receptors (Aleman et al., 2016). The transduction of signals from receptors to the nucleus during NET induction engages many typical mediators including the spleen tyrosine kinase (Syk)/Src kinase family (Nanì et al., 2015), protein kinase C (PKC) (Neeli and Radic, 2013), extracellular signal–regulated kinases (ERK1/2) (Hakkim et al., 2011; Keshari et al., 2012; DeSouza-Vieira et al., 2016), phosphoinositide 3-kinase (PI3K) (Behnen et al., 2014; DeSouza-Vieira et al., 2016), and NADPH oxidase (Nishinaka et al., 2011; Parker et al., 2012). During netosis, the nuclear envelope is decomposed, the chromatin is decondensed and the DNA is complexed with different proteins released from ruptured granules. The cell membrane is subsequently ruptured and the NETs are released from the cells. Cytoplasmic proteins are rarely found in the NET structure, confirming that the protein/DNA complexes in NETs do not form via a random process (Urban et al., 2009).

*C. albicans* is readily recognized by neutrophils and the aspartic proteases produced by this microbe are chemotactic agents for neutrophils (Gabrielli et al., 2016) and are probably involved in their modulation via ROS generation (Hornbach et al., 2009). For the inactivation of fungal cells, neutrophils can also utilize netosis in which calprotectin seems to be the main killing component of NETs (Urban et al., 2006). Moreover, as the hyphal form of *C. albicans* appears to be too bulky to be phagocytosed, it has been proposed that NET release is activated and regulated by the size and type of morphological form of *C. albicans* and is therefore the ideal mechanism to prevent invasion by the filamentous forms of this pathogen (Branzk et al., 2014; Johnson et al., 2016). Furthermore, the major components of the *C. albicans* cell wall can be involved in the activation of netosis pathways. β-Glucans, which are recognized by the Dectin-1 or CR3 receptors of neutrophils (Li X. et al., 2011; Gazendam et al., 2014; Johnson et al., 2016) cause NET release, probably via a ROS-independent mechanism (Byrd et al., 2013; Nanì et al., 2015). However, the detailed roles of each type of *C. albicans* surface or secreted component in NET formation are still poorly understood.

In our current study, we aimed to determine the functions of the main components of the *C. albicans* cell wall, including β-glucans, mannans and adhesive proteins, and Saps, in the process of NET formation. We found evidence that each major class of compounds isolated from the fungal cell wall can activate netosis and revealed for the first time that the *C. albicans* Saps play an important role as a NET-release trigger. We identified the main receptors involved for each class of compounds that was examined in the present study and identified that the CD11b receptor interacts with Sap6, one of the main triggers of netosis. Taken together, our current findings have enabled us to propose a scheme for the signaling pathways involved in the induction of netosis by fungal pathogens.

## Materials and methods

### Yeast strains and culture

The main experiments were performed using *C. albicans* strain ATCC10231 purchased from the American Type Culture Collection (Manassas, VA). The role of *C. albicans* Saps in netosis was also examined using the *C. albicans* strains listed in Table 1. *C. albicans* was grown in YPD medium (1% yeast extract, 2% soybean peptone, and 2% glucose) for 16 h at 30°C in an orbital incubator to the stationary phase. For the isolation of cell wall components, *C. albicans* hyphae were obtained by inoculation of the stationary-phase cells into RPMI-1640 medium (PAA Laboratories GmbH, Pasching, Austria) followed by incubation for 72 h at 37°C with constant shaking. To compare the NET release by two morphological forms of *C. albicans*, the same number of fungal cells was used in two sets. The yeast-like form was obtained directly from the YPD culture, after washing and re-suspending the cells in the PBS buffer, whereas the second set was incubated under hypha-inducing conditions (RPMI, 3 h at 37°C), in which the cell number was kept nearly the same but the cell morphology changed. The initial cell count was determined by OD measurement at 600 nm, performed for the diluted liquid culture and assuming that the OD_600_ value equal to 1 corresponds to 3 × 10^7^ cells/ml.

**Table 1:** *C. albicans* strains used in this study.

**Strain**	**Genotype**	**References**
ATCC 10231	Wild type	
CAI-4	Prototrophic wild type	Fonzi and Irwin, 1993; Murad et al., 2000
*sap*Δ*/*Δ *1/2/3*	*sap1*Δ*::hisG/sap1*Δ*::hisG* *sap2*Δ*::hisG/sap2*Δ*::hisG* *sap3*Δ*::hisG/sap3Δ:hisG*	Negri et al., 2011
*sap*Δ*/*Δ *4/5/6*	*sap6*Δ*::hisG/sap6*Δ*::hisG* *sap4*Δ*::hisG/sap4*Δ*::hisG* *sap5*Δ*::hisG/sap5Δ:hisG*	Sanglard et al., 1997
*sap8*Δ*/*Δ	*Δsap8::hisG/Δsap8::hisG-*URA3-hisG	Puri et al., 2012
*sap*Δ*/*Δ *9/10*	*sap10*Δ*::hisG/sap10*Δ*::hisG* *sap9*Δ*::hisG/sap9Δ:hisG*	Albrecht et al., 2006

### Neutrophil isolation

Human polymorphonuclear cells (PMNs) were isolated from EDTA-treated whole-blood samples obtained from healthy donors via the Regional Blood Donation Center (Krakow, Poland), which complies with the requisite confidentiality assurances for human participants. Whole blood (20 ml) was collected into 50 ml tubes containing EDTA at a final concentration of 5 mM. Samples were maintained at room temperature throughout the isolation procedure. The tubes were centrifuged at 300 × g for 15 min, the plasma layer was removed, and the bottom layer containing leukocytes and erythrocytes was restored to the original volume of 20 ml with phosphate-buffered saline (PBS) (Sigma-Aldrich, St. Louis, MO) and gently mixed by inverting the tube four times. The cell suspension was carefully layered on 10 ml Pancoll (PAN-Biotech GmbH, Aidenbach, Germany) and centrifuged at 300 × g for 30 min. The supernatant was removed and 20 ml of 1% polyvinyl alcohol (Sigma-Aldrich) was gently mixed with the suspension by inverting four times. The tubes were allowed to stand for 20 min at room temperature to enable erythrocyte sedimentation. The supernatant containing the PMNs was then collected into a fresh tube and centrifuged at 110 × g for 5 min. The supernatant was next removed and the erythrocytes in the cell pellet were lysed by the addition of 1 ml of sterile H_2_O for 30 s. An equivalent of 2x-concentrated PBS was added to stop the lysis reaction. The volume of cell suspension was filled to 10 ml by PBS and centrifuged at 110 × g for 5 min. The supernatant was removed, the cell pellet was resuspended in 1 ml RPMI-1640 medium without phenol-red (PAA Laboratories). The neutrophil purity was assessed routinely by forward- and side-scatter flow cytometric analyses and the rapid Romanowsky stain of cytospins followed by differential count of more than 700 cells, using optical microscopy. This neutrophil preparation method yields a >95% pure population of the cells.

### Isolation of mannans and glucans from the *C. albicans* cell wall

Mannans were isolated from the *C. albicans* cell wall using the method previously described by Kocourek and Ballou (1969). Briefly, this procedure is based on the precipitation of the mannans released from cell walls obtained after autoclaving the fungal cells for 3 h at 121°C in 20 mM citrate buffer, pH 7.0. This precipitation of mannans from the supernatant was then repeated twice with Fehling's reagent obtained just before the experiment by mixing solution A (69,278 g/l of CuSO_4_ × 5 H_2_O) and B (346 g/l of potassium-sodium tartrate and 100 g/l of NaOH). The remaining cell pellet, after homogenization, was used to isolate the glucans by heating the sample for 2 h in a water bath with isopropyl alcohol (w/v ratio of 1:4) under reflux. The excess reagent was removed by washing three times with acetone. Proteinaceous contaminations which can remain in the polysaccharide preparations were removed by hydrolyzing with 250 μg of proteinase K (Sigma-Aldrich) for 4 h at 55°C in 20 mM Tris-HCl buffer, pH 8.0 containing 20 mM NaCl, 2 mM MgCl_2_, 1 mM CaCl_2_, 0.1 mM DTT (Liu et al., 2008). Possible contamination of glucan and mannan fractions with other cell surface proteins was tested using the method of Bradford (1976). The concentration of mannans or glucans was determined using the phenol-sulfuric acid method (Dubois et al., 1951; Masuko et al., 2005), modified for 96-well microtiter plates (Sarstedt, Nűmbrecht, Germany). About 200 ng of glucans and 80 ng of mannans could be obtained from 10^5^ cells.

### Isolation of *C. albicans* cell wall proteins

Cells from the hyphal form of *C. albicans* (0.4 g wet weight) were washed three times with 50 mM phosphate buffer, pH 6.0, twice with 10 mM Tris buffer with 0.9% NaCl, pH 7.4, and once with 50 mM Tris buffer, pH 7.5. The extracts of cell wall proteins were prepared from cells by treatment with β-1,3-glucanase (Quantazyme; Qbiogene, Carlsbad, CA; Crowe et al., 2003). Quantazyme (200 U) was added to the cell suspension in 1 ml of 50 mM Tris buffer, pH 7.5 containing 40 mM 2-mercaptoethanol and protease inhibitor cocktail (Roche, Penzberg, Germany). The cells were then incubated at 37°C for 3 h with gentle shaking. Trypan Blue staining was used to verify that at least 95% of the *C. albicans* cells remained viable after this treatment. The treated cells were allowed to settle and after cell pellet removal, the released cell surface proteins in the supernatant were purified from DNA by ion chromatography on MonoQ-Sepharose (GE Healthcare/Pharmacia, Uppsala, Sweden) equilibrated in 20 mM Tris-HCl buffer, pH 8.0. The protein fraction was then eluted using a gradient of NaCl (0–0.4 M) to receive cell wall protein mixture (CWP). The purification of Als3 and enolase was performed according to the method described by Seweryn et al. (2015).

### Recombinant Sap production and protein fluorescent labeling

All 10 Sap isoenzymes used in the experiments were obtained according to the method described previously (Aoki et al., 2011; Rapala-Kozik et al., 2015), after their overexpression in the *Pichia pastoris* system (Invitrogen, Waltham, MA). The homogeneity of the purified proteins was checked by sodium dodecyl sulfate-polyacrylamide gel electrophoresis (SDS-PAGE) and their proteolytic activities were assayed on the boron-dipyrromethene FL casein substrate (Invitrogen) in 0.1 M buffers at pH values corresponding to the highest activities of the enzymes (Aoki et al., 2011). Purified Sap6 was labeled using 5/6-carboxyfluorescein succinimidyl ester (NHS-Fluorescein; Pierce Biotechnology, Rockford, IL). For the other protein samples (200 μl), prepared in labeling buffer (20 mM sodium phosphate, 150 mM sodium chloride, pH 8.0), a 20-fold molar excess of freshly prepared solution (10 mg/ml) of NHS-fluorescein in dimethylsulfoxide (DMSO) was added. The reactions were performed for 2 h on ice. Non-reacted NHS-fluorescein was removed using a Dye Removal Column (Pierce Biotechnology) by centrifugation for 30 s at 1,000 × g. The concentration of labeled protein was determined with the bicinchoninic acid (BCA) method. Protein preparations were stored at 4°C.

### NET visualization

Microscope slides were cleaned with isopropyl alcohol and coated with 0.01 mg/ml of poly-L-lysine at 4°C overnight. Then, 2.2 × 10^5^ neutrophils in 80 μl RPMI-1640 medium were seeded on the slides and incubated for 30 min at 37°C, 5% CO_2_ for cell attaching. Whole *C. albicans* cells or selected stimulants including isolated glucans, mannans, isolated mixture of cell wall proteins (CWP) or purified proteins (enolase, Als3, Saps) were then added in the volume of 20 μl of RPMI at various range of concentrations. Neutrophils, treated for 3 h at 37°C, 5% CO_2_ with 25 nM PMA (Sigma-Aldrich) were used as a positive control. After incubation, SytoxGreen dye (Molecular Probes, Eugene, OR) was added to each cell sample at a final concentration of 1 μM. Samples were visualized under a fluorescence microscope (Nikon Eclipse-Ti). NET production was also visualized using antibodies against myeloperoxidase or elastase (Abcam, Cambridge, UK).

### Monitoring of neutrophil interaction with *C. albicans* cells or their surface components via quantification of the NET response

Twelve-well microplates (Greiner Bio-One, Germany) were coated with poly-L-lysine at 4°C overnight. An aliquot of 1.5 × 10^6^ neutrophils in 900 μl RPMI-1640 medium was added to each coated well and the cells were incubated at 37°C in 5% CO_2_ for 30 min to enable attachment to the surface. Whole *C. albicans* cells, as well as stimuli factors including isolated glucans, mannans, whole CWP mixtures or purified proteins (enolase, Als3, Saps) were added in a volume of 20 μl of RPMI at a selected range of concentrations. The cells were then further incubated at 37°C in 5% CO_2_ for 3 h. Cell treatment with PMA was used as a positive control. After incubation, the cells were washed three times with PBS. A 400 μl aliquot of micrococcal nuclease (MNase) at a concentration of 1 U/ml (Roche) was then added and the cells were incubated at 37°C for an additional 20 min to digest any released DNA. The nuclease activity was stopped by the addition of 5 mM EDTA. The microplate was finally centrifuged for 5 min at 300 × g at 4°C and the collected supernatants were once again centrifuged to remove any debris. SytoxGreen was added to the clarified solutions at a final concentration of 1 μM and 50 μl of each sample was transferred into a 96-well microplate to measure fluorescence (excitation at 495 nm, emission at 525 nm) using a Synergy H1 microplate reader (Biotek, Winooski, VT).

### Test of neutrophil viability

Neutrophil apoptosis was analyzed using an annexin V-FITC Apoptosis Detection Kit (ab14085, Abcam). Briefly, the neutrophils (2.2 × 10^5^) were incubated with *C. albicans* cells at a multiplicity of infection (MOI) of 0.01–10, at 37°C in 5% CO_2_ for 3 h. The cell suspensions were then washed three times with PBS and resuspended in 500 μl of PBS containing annexin V-FITC and propidium iodide (PI). Samples were further incubated for 5 min in the dark at room temperature and analyzed by flow cytometry using a FACS BD LSR II cytometer (BD, San Jose, CA) with a FITC signal detector for annexin V-FITC binding and phycoerythrin signal detector for PI staining.

### Analysis of Sap6 interaction with neutrophil surface

Neutrophils (2.2 × 10^5^) in 100 μl of RPMI-1640 medium were transferred into a 96-well high binding black microplate (Greiner Bio-One, Kremsmünster, Austria) and left at 37°C for 30 min to attach. The plate was then washed three times with 0.05% bovine serum albumin (BSA) in PBS and further incubated in this solution for 30 min at 37°C to coat the unoccupied well surface. After further washing of the cells with PBS, 100 μl aliquots of fluorescently labeled Sap6 at various concentrations were added to the wells. The plate was then incubated for 1 h at 37°C and washed three times with PBS. Fluorescence was measured (465/525 nm) in fresh PBS using the Synergy H1 microplate reader.

For microscopic analysis of neutrophil interaction with Sap6, 10^5^ neutrophils suspended in RPMI medium were settled for 30 min at 37°C on glass coverslips pre-coated with poly-L-lysine (1 mg/ml). Cells were incubated with fluorescein-labeled Sap6 for 1 h at 37°C, washed with PBS and fixed with 3.7% formaldehyde for 10 min at room temperature. Glass slides were then washed twice with PBS. Mounting medium Fluoromount-G (Southern Biotech) was added to the preparations. The confocal microscopy images were collected using an TCS SP5 II microscope (Leica).

### Identification of the neutrophil receptors and signal mediators involved in netosis

For investigation of the role of selected receptors or signal mediators in netosis, neutrophils were preincubated with specific antibodies or inhibitors prior to stimulation with *C. albicans* factors. Neutrophils (1 × 10^6^) were preincubated for 30 min at 37°C in RPMI-1640 medium with 1 μg/ml of blocking antibodies directed against TLR2, TLR4, Dectin-1, CD14 (Invivogen, Toulouse, France), CD11a, CD11b, CD16, CD18 (BioLegend, San Diego, CA, USA) or isotype control antibody—IgG (Abcam). For the analysis of selected signaling pathways, neutrophils (1 × 10^6^) were preincubated for 30 min at 37°C in RPMI-1640 medium with 30 μM of Syk inhibitor (piceatannol; Sigma-Aldrich), 10 μM of Src inhibitor (PP2; Calbiochem, Darmstadt, Germany), 25 μM of PI3K inhibitor (LY29004; Calbiochem), or 10 μM of ERK inhibitor (UO126; Cell Signaling Technology, Beverly, MA). To inhibit ROS-dependent netosis pathways, neutrophils were preincubated for 30 min at 37°C in RPMI-1640 medium with 10 μM DPI (Sigma-Aldrich), an NADPH oxidase inhibitor. Phagocytosis of neutrophils was blocked by pretreatment of the cells with 5 μM of cytochalasin D (CytD, Sigma-Aldrich) for 15 min at 37°C. After removal of the blocking agent by cell washing, neutrophils were treated with the indicated stimulants. Untreated neutrophils were used as controls.

### Analysis of ERK1/2 activation

ERK1/2 activation was analyzed using an ERK 1/2 (pT202/Y204 + Total) ELISA Kit (ab176660, Abcam). Neutrophils (1 × 10^5^) were incubated in RPMI-1640 medium with selected Saps at a concentration of 1 ng/ml for 1 h at 37°C in 5% CO_2_. The cells were then washed three times with PBS and lysed in lysis buffer containing phosphatase and protease inhibitors provided by the manufacturer. Fifty microliters of aliquots of each lysate were transferred into the wells of antibody pre-coated strips and 50 μl of a solution containing antibodies coupled with peroxidase was added. The strips were then incubated for 1 h at room temperature with continuous gentle shaking. The wells were then washed three times with the provided buffer and the plate was incubated for an additional 15 min following the addition of 3,3′,5,5′-tetramethylbenzidine (TMB). The enzymatic reaction was stopped by acidification and the optical density (OD) was recorded for each well at 450 nm using the Synergy H1 microplate reader.

### Statistical analysis

Experiments were performed at least three times in duplicate using neutrophils from different donors on different days. Statistics were performed by one-way ANOVA analysis followed by Turkey's *post-hoc* test, using GraphPad Prism 7 software (GraphPad Software, La Jolla, CA, USA). Differences of *p* < 0.05 were considered significant.

## Results

### The main virulence factors of *C. albicans* are involved in triggering NET formation

**Figure 1:**
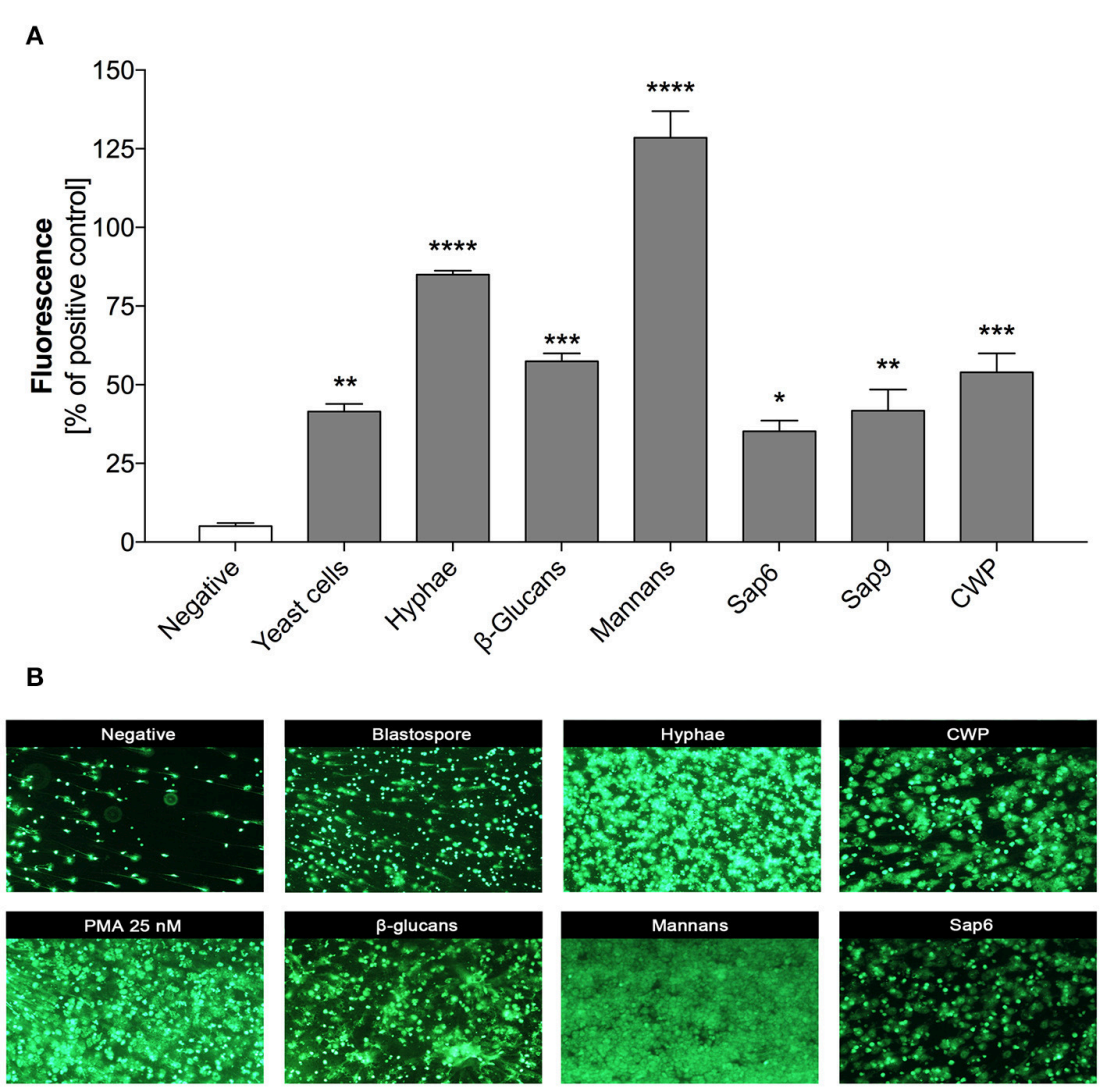
NET release in response to selected *C. albicans* extracellular components. Neutrophils (2.2 × 10^5^) in RPMI medium were treated for 3 h with solutions of purified *C. albicans* extracellular components: glucans, mannans, cell wall proteins (CWP) and Saps or whole *C. albicans* cells, pre-propagated for 3 h at 37°C under different conditions (YPD or RPMI), to obtain representations of two fungal morphological forms (yeasts and hyphae, respectively) at an MOI of 1:1, as was described in detail in Materials and Methods section. Neutrophils treated with 25 nM PMA were used as a positive control whereas untreated cells served as a negative control. **(A)** Extracellular DNA from neutrophils was partially digested with micrococcal nuclease (MNase) and quantified. The fluorescence intensity measured after Sytox Green treatment was used to quantify NET release relative to the positive control. The results shown are derived from three independent experiments performed in duplicate. Data represent the means (relative to PMA) ± SEM. Asterisks denote a statistically significant difference between the samples and negative control (^*^p < 0.05, ^**^p < 0.01, ^***^p < 0.005, ^****^p < 0.001, ns—no significance). **(B)** Extracellular DNA was visualized by fluorescence microscopy after staining with SytoxGreen. The results shown are a representative of five independent experiments, each performed in triplicate.

Some recent studies have described the different involvement of two morphological forms of *C. albicans* cells in NET release by neutrophils upon contact with fungi, with a stronger effect found for the hyphal form (Urban et al., 2006; Branzk et al., 2014; Johnson et al., 2016). Although, all *C. albicans* cells are equipped with principal virulence factors such as cell-wall polysaccharides (glucans, mannans), surface proteins and proteases (either surface-localized or secreted to the surrounding environments), the specific profiles and proportions of these factors are different and are dependent on the location and extent of infection (Naglik et al., 2008). To compare their efficiency in NET release, we used purified virulence factors isolated from the filamentous form of *C. albicans*. The effects on NET release after 3 h of neutrophil treatment were detected by SytoxGreen staining and were compared with the results observed during neutrophil contact with 25 nM phorbol myristate acetate (PMA), a potent inducer of NETs, and corresponding to neutrophil-contacted whole fungal cells at an MOI 1:1. Our results (Figure 1) confirmed previous observations in the literature on the higher NET response levels to the filamentous forms of *C. albicans* as compared to planktonic cells. The most intense signal was detected with compounds that form the core of the fungal cell wall, i.e., mannans and glucans, and produced signals even exceeding the PMA effect (130 and 60%, respectively). Weaker responses of neutrophils were found for proteinaceous fungal cell surface compounds, mainly mannoproteins (up to 60%) involved in the adhesion of *C. albicans* cells, as well as the surface-attached protease Sap9 (48%). The effect of Sap9 was comparable to that of Sap6 (35%), which is the aspartic protease secreted to the environment by the hyphal form of *C. albicans*.

### NET formation is determined by the progress of *C. albicans* infection

**Figure 2:**
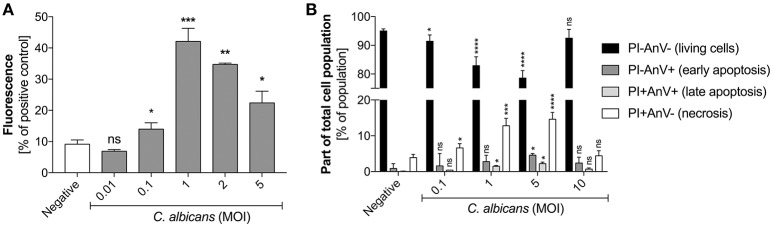
**(A)** Generation of NETs by *C. albicans* at different MOI levels. **(B)** Neutrophil viability upon contact with *C. albicans*. **(A)** Neutrophils (2.2 × 10^5^) were incubated with *C. albicans* cells at different MOI levels for 3 h to induce netosis. After incubation, the amount of released DNA was determined using MNase for partial NET digestion followed by staining with SytoxGreen. Unstimulated neutrophils were used as a negative control; a 25 nM PMA solution was assumed to induce a maximal level of NET release. Results from three independent experiments (in triplicate) are shown. Error bars represent the mean ± SEM and are expressed as a percentage ratio of the tested sample signal relative to the PMA-induced control. Asterisks denote statistical significance relative to the negative control (^*^p < 0.05, ^**^p < 0.01, ^***^p < 0.005, ^****^p < 0.001, ns—not significant). **(B)** After incubation with *C. albicans* cells, neutrophils were labeled with annexin V-FITC and propidium iodide and analyzed by flow cytometry. The results are presented as a percentage ratio of the signal detected for whole cell population and showing no cell death (PI-AnV-), early apoptosis (PI-AnV+), and late apoptosis (PI+AnV+). Cells stained only with propidium iodide (PI+AnV-) represent the necrotic or NET-forming cells. For each sample, data were collected for 100,000 neutrophils. Data are presented as mean ± SEM of three independent experiments. Asterisks denote statistical significance relative to the negative control (^*^p < 0.05, ^**^p < 0.01, ^***^p < 0.005, ^****^p < 0.001, ns—not significant).

The previously reported data suggesting the impact of host proteins or the pathogen size on the quantity of NET release by neutrophils in contact with *C. albicans* (Branzk et al., 2014), and their possible influence on microbial biofilm formation (Johnson et al., 2016), prompted us to assess a possible correlation between NET release and the progress of infection. We simulated infection progression by increasing the MOI (multiplicity of infection) of the contacting cells. For more precision and to eliminate the effect of biofilm formation we used planktonic cells that started to germinate during the time of contact with neutrophils (3 h, 37°C, RPMI). At a low amount of *C. albicans* cells (up to a 1:1 MOI), the alarmed neutrophils responded by increasing NET production, suggesting an important role of netosis at the initial infection stage (Figure 2A). However, further increases in the amount of fungal cells in contact with neutrophils (an MOI above 2:1) resulted in a declining NET release.

To test whether this observation was the result of different types of neutrophil death, we assessed neutrophil viability after exposure to *C. albicans* cells by FACS analysis (Figure 2B). The results indicated that the number of propidium iodide positive cells (PI+) correlated with netosis at different MOI values. At an MOI of 1:1 and 1:5 the percentage of PI+ cells reached the highest level, but at the MOI of 1:10 this trend was reversed, i.e., the number of living cells increased and NET production was not observed. This finding suggests the involvement of other mechanisms of defense against fungal cells.

### Glucans and mannans trigger NET release by different mechanisms involving reactive oxygen species

**Figure 3:**
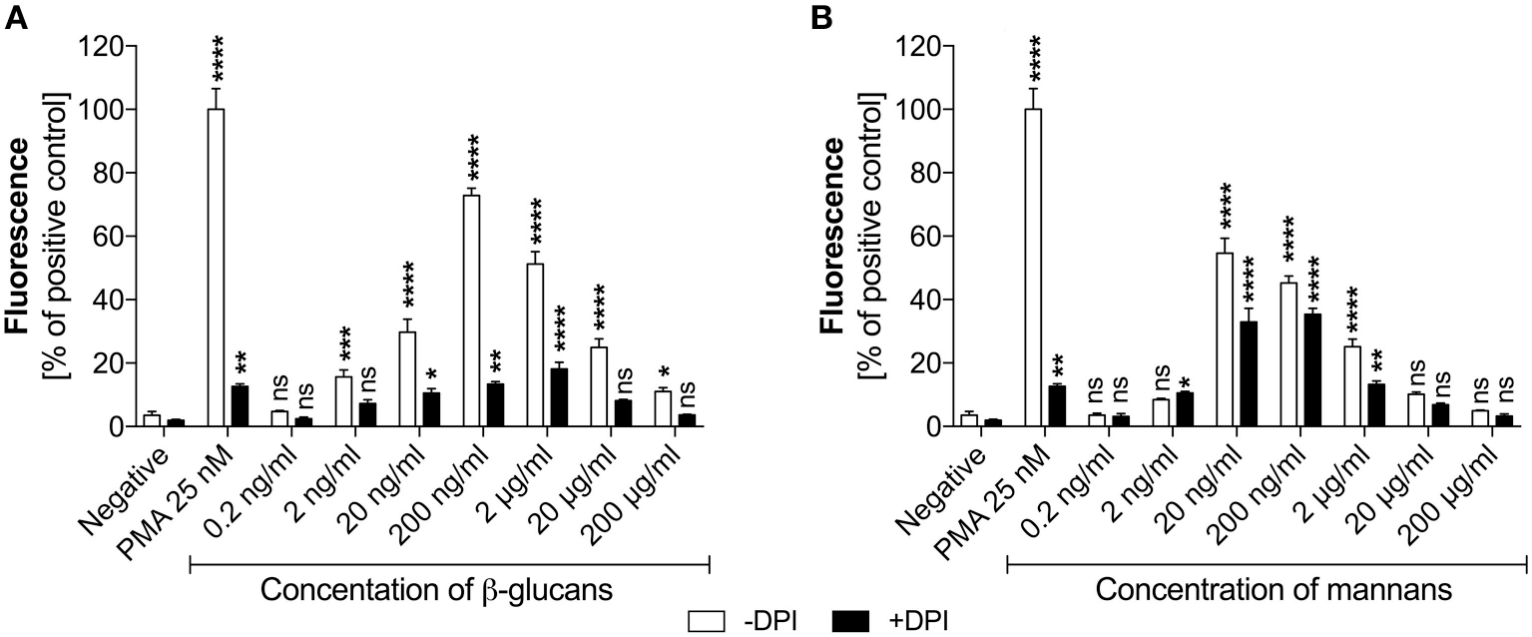
Generation of NETs triggered by glucans and mannans. Neutrophils (2.2 × 10^5^) were incubated for 3 h with isolated *C. albicans* glucans **(A)** or mannans **(B)** at the indicated concentrations to induce netosis. Cells with active NADPH oxidase (−DPI) or with this enzyme inhibited by 5 μM DPI (+DPI) were used. The amount of released DNA was determined fluorometrically after SytoxGreen staining, as described above. The negative control was unstimulated neutrophils, while treatment with 25 nM PMA solution was assumed to induce a maximal NET release. The results shown are representative of three independent experiments performed in duplicate. Data represent the means ± SEM and are shown as percentage ratio of the signal for the tested sample relative to the PMA-induced control. Asterisks denote statistical significance relative to the negative control (^*^p < 0.05, ^**^p < 0.01, ^***^p < 0.005, ^****^p < 0.001, ns—not significant).

Based on the identified effects of *C. albicans* surface components on NET release, a deeper analysis was conducted to determine the receptor involvement and ROS-dependent nature of these responses. As the type and amount of glucans or mannans on the *C. albicans* cell surface may change during infection, depending on host cell response, we first assessed whether there was any correlation between NET production and polysaccharide concentration (Figure 3). The amount of glucans and mannans used in these experiments corresponded to the estimated amounts on the fungal cell surface at an MOI of 1:1.

In the analysis, the presence of glucans at a concentration of up to 200 ng/ml (Figure 3A) led to the release of NETs in a dose-dependent manner. Surprisingly, further increases in the glucan concentration resulted in a significant decrease in the netosis yield. The most efficient NET production level, i.e., about 70% of total amount of DNA released after PMA stimulation, was observed for the glucan concentration of 200 ng/ml that corresponds to an MOI of 1:1. Moreover, this glucan-triggered process seemed to involve a ROS-dependent pathway, in which NADPH oxidase is the key mediator of NET release. This was evidenced by the effect of treating neutrophils with the NADPH oxidase inhibitor diphenyleneiodonium chloride (DPI) prior to glucan usage. DPI exposure caused a significant reduction in the neutrophil response, below 20% of the positive control level. This effect was similar to that detected using neutrophils treated with PMA in the presence of DPI, suggesting that this process is strongly ROS-dependent. A similar pattern of netosis activation was observed with mannans (Figure 3B). The maximal efficiency of NET production, about 60% of the positive control level, was detected at a mannan concentration of only 20 ng/ml. Notably, a further increase in the mannan concentration also caused a lower NET response. However, the netosis pathway triggered by exposure to mannans seemed to differ from that induced by glucans. The inhibition of NADPH oxidase by DPI had little effect on NET formation in the presence of mannans, with a minor blocking impact of DPI on DNA release. This finding suggests that mannans act predominantly, but not exclusively, through an ROS-independent netosis pathway.

**Figure 4:**
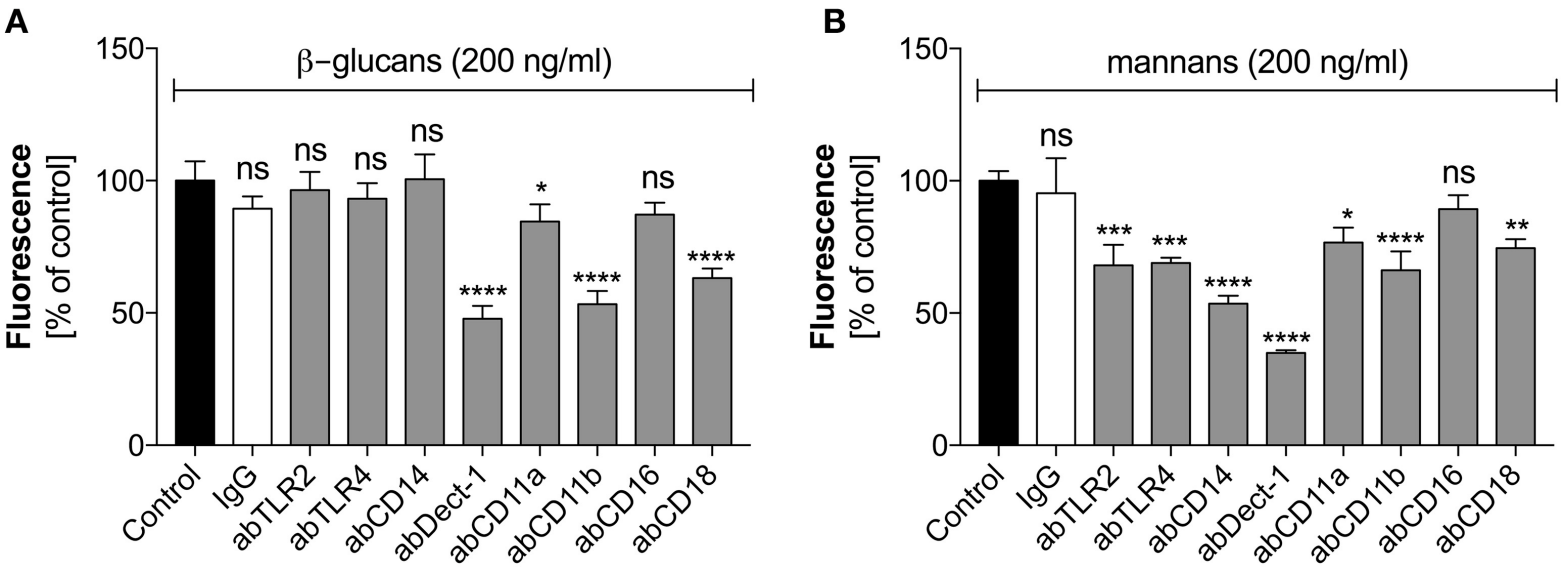
The role of neutrophil receptors in the activation of netosis by glucans and mannans. Neutrophils (2.2 × 10^5^) were preincubated with selected antibodies (**ab**, 1 μg/ml) against the indicated surface receptors and netosis was then induced for 3 h by incubation with glucans **(A)** or mannans **(B)** at a concentration of 200 ng/ml. IgG antibody was used as the isotype control. The amount of released DNA was determined using SytoxGreen staining and fluorimetric detection, as described above. The results shown are derived from three independent experiments performed in duplicate. Data are represented by the means ± SEM and are expressed as percentage ratio of the detected signal for the tested sample relative to the control sample. Asterisks denote statistical significance relative to the negative control (^*^p < 0.05, ^**^p < 0.01, ^***^p < 0.005, ^****^p < 0.001, ns—no significance).

These two types of *C. albicans* polysaccharide cell wall components are recognized by various neutrophil receptors. We therefore attempted to identify the receptors involved in the activation of netosis by glucans (Figure 4A) and mannans (Figure 4B). Specific antibodies were used to block a range of receptors prior to stimulation with glucans at concentration of 200 ng/ml or mannans at concentration of 20 ng/ml. The decrease in NET response would then indicate the receptor participation in netosis activation by neutrophils. As shown in Figure 4A, an active role in the glucan-triggered process was shown for the CD369 (Dectin-1), CD11b (Mac-1), and CD18 receptors, in which CD11b and CD18 were found to be cooperating receptors. Antibody blocking of these receptors led to a 50–60% decrease in the NET response. A lowering in NET production was also observed for a CD11a receptor block, where incubation with a specific antibody decreased the extracellular DNA release to about 80–85% of the initial level.

In the case of mannan-triggered netosis, it was found to be mediated by many different surface receptors (Figure 4B), principally the CD369 (Dectin-1), CD11b (Mac-1), and CD14 receptors. CD14 is a co-receptor for TLR4, which, together with TLR2, also played a role in mannan recognition by neutrophils. A slight effect was also noted when blocking the CD18 and CD11a receptors. The considerable differences in the receptors involved in mannan-triggered netosis may explain the possible mixed mechanisms, i.e., ROS-independent and ROS-dependent, underlying the NET response to these molecules.

### *C. albicans* surface proteins are less effective in activating netosis

**Figure 5:**
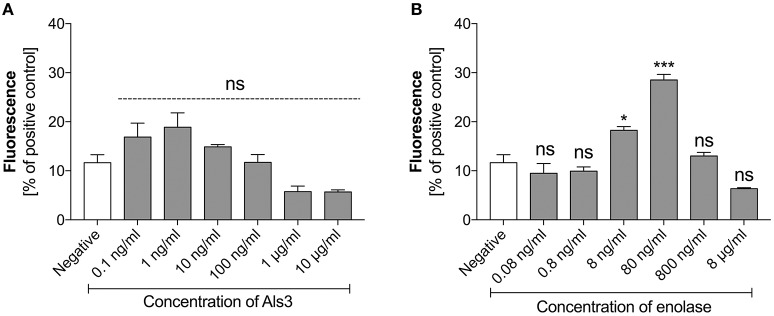
Response of neutrophils to stimulation with Als3 and enolase. Neutrophils (2.2 × 10^5^) were incubated with selected concentrations of Als3 **(A)** or enolase **(B)** for 3 h to induce netosis. The amount of released DNA was determined fluorimetrically after SytoxGreen staining, as described above. The results shown are derived from three independent experiments performed in duplicate. Data are presented as the means ± SEM and expressed as a percentage ratio of the signal for the tested sample relative to the positive, PMA-induced control. Asterisks denote statistical significance relative to the negative control (^*^p < 0.05, ^***^p < 0.005, ns—not significant).

As identified in our earlier analyses, *C. albicans* surface proteins also participated in NET production by neutrophils in contact with *C. albicans* cells. To determine the ability of these surface proteins to activate NET release we purified those that have been commonly detected during *C. albicans* host infections. These included Als3—one the main candidal adhesins and a representative of the glycosylphosphatidylinositol (GPI)-anchored surface mannoproteins, and enolase, the most prominent “moonlighting” protein that has well-confirmed adhesive function (Liu and Filler, 2011; Karkowska-Kuleta and Kozik, 2014) and is a widely used antigen in serological tests for candidal infection (Li Wq. et al., 2011; Shibasaki et al., 2013). Both proteins were tested for their ability to stimulate NET formation in a concentration-dependent manner. Als3 did not show any significant influence on NET release (Figure 5A). In contrast, enolase showed a positive correlation with the NET response, but only within a narrow concentration range (Figure 5B). Enolase caused a maximal effect at 80 ng/ml, releasing NETs at 30% of the positive control level. Again, a further increase in the enolase concentration in contact with neutrophils inhibited netosis.

### Aspartic proteases of *C. albicans* trigger netosis with high potency

#### Sap production mutants are less effective in activating NETs

**Figure 6:**
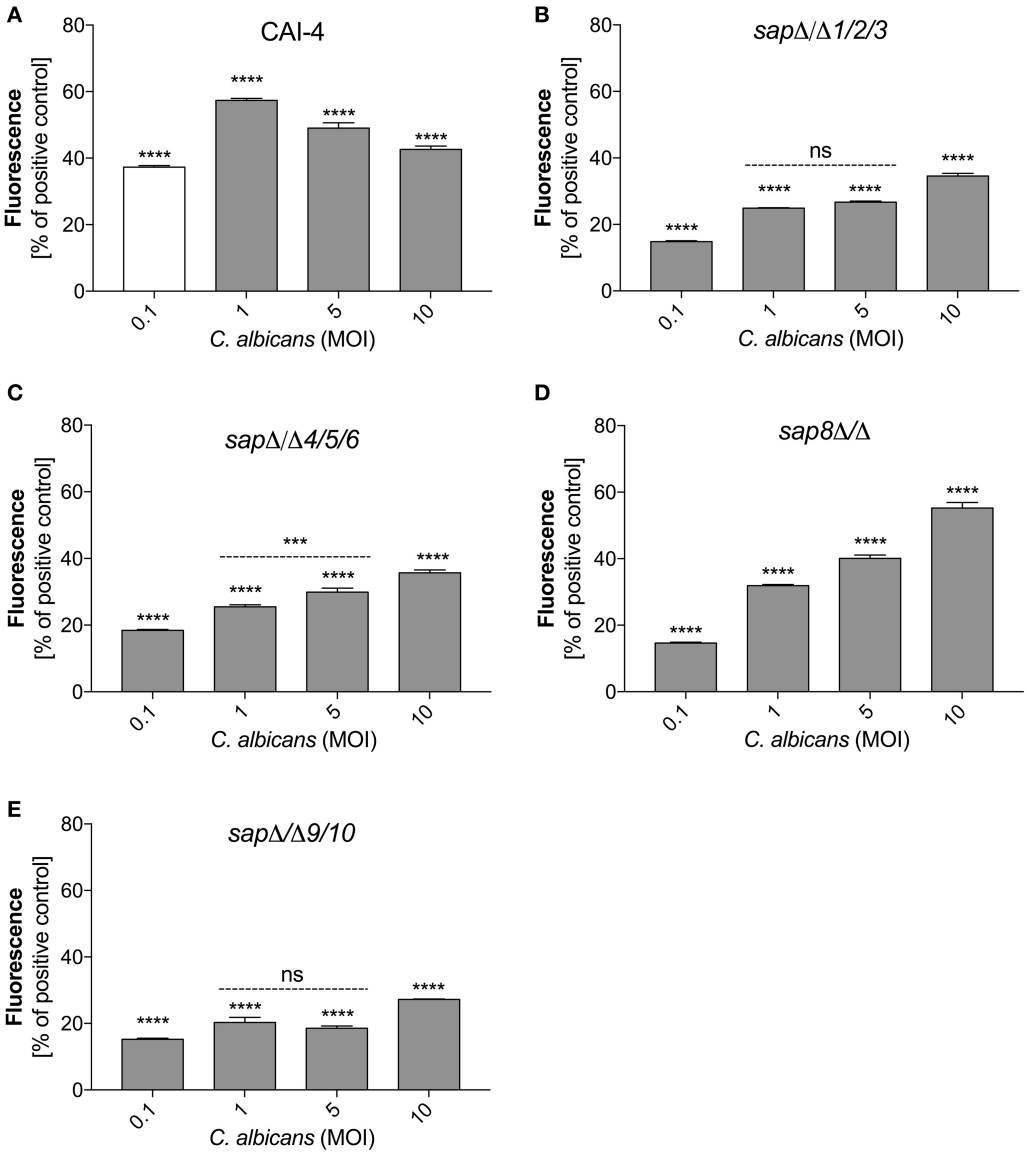
Variable Sap expression regulates NET release. Neutrophils (1.5 × 10^6^) were incubated with cells from the indicated strains of *C. albicans* at different MOI level for 3 h to induce netosis. Strains with deletions in the *SAP1/2/3* (*sap*Δ*/*Δ*1/2/3*) **(B)**, *SAP4/5/6* (*sap*Δ*/*Δ*4/5/6*) **(C)**, *SAP8* (*sap8*Δ*/*Δ) **(D)**, and *SAP9/10* (*sap*Δ*/*Δ*9/10*) **(E)** genes were used, as well as reference cells (CAI-4) **(A)**. The results shown are representative of three independent experiments performed in duplicate. Data are the means ± SEM and are expressed as percentage ratio of the signal for the tested sample relative to the positive PMA-induced control. Asterisks denote statistical significance relative to the positive control (^***^p < 0.005, ^****^p < 0.001, ns—not significant).

To verify the role of secreted and surface localized aspartic proteases of C. *albicans* in the activation of netosis, we used *C. albicans* strains harboring selected Sap group gene deletions (Figure 6). We used the *C. albicans* CAI-4 strain as the reference. CAI-4 responded depending on the MOI in the same way as the wild-type ATCC10231 strain. We observed the highest efficiency of NET production at an MOI of 1:1, corresponding to 60% of the positive control level with PMA exposure. All of the mutant strains of *C. albicans* could initiate netosis in an MOI-dependent manner but they responded with different efficiencies depending on the deleted protease. The quantity of released DNA by neutrophils in contact with mutant strains at an MOI of 1:1 was significantly lower than the reference strain. In addition, the trend for the observed changes in the mutant strains was different—the amount of released DNA still increased with an increasing MOI. This could suggest that the trigger that inhibited netosis in the wild type fungal cells was not sufficient in the mutant cells lacking protease activity. *C. albicans* cells deprived of the aspartic proteases Sap9 and Sap10 (*sap*Δ/Δ9/10), that are GPI-anchored in the cell wall, showed the lowest NET response, reaching only about 25% of the positive control cells (neutrophils treated with PMA) at an MOI of 1:10. The deletion of Sap1-Sap3 (*sap*Δ/Δ1/2/3) and Sap4-Sap6 (*sap*Δ/Δ4/5/6) resulted in NET generation below 40% of the positive control levels at an MOI of 1:10. However, the lack of Sap8 seemed to have no effect on NET production and at an MOI of 1:10 these mutant cells showed a response similar to that of wild-type cells. Comparing these results with the findings at an MOI of 1:1 it could be concluded that the tested proteases have important roles in netosis activation.

#### Purified Saps activate NET release in a dose dependent manner, partially via a ROS-dependent pathway

**Figure 7:**
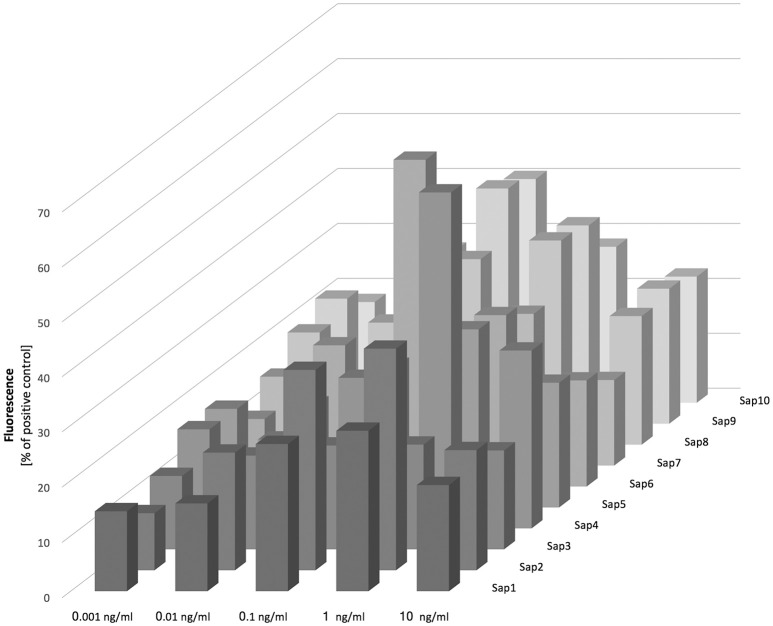
Release of NETs in response to Saps. Neutrophils (2.2 × 10^5^) were incubated for 3 h with isolated Saps (Sap1-10) within the 0.001–10 ng/ml concentration range to induce netosis. The amount of released DNA was determined fluorimetrically after SytoxGreen staining, as previously described. Data are the means ± SEM from two experiments and are expressed as a percentage ratio, as described above.

To verify the role of the aspartic proteases in triggering netosis, we treated neutrophils with purified Sap proteins at a wide range of concentrations (0.001–10 ng/ml; Figure 7). NET release was then measured by the detection of excreted DNA using SytoxGreen, and was also confirmed by microscopic detection of elastase or myeloproxidase to exclude the effects of cell membrane disruption by fungal proteases (data not presented). Moreover, the influence of proteolytic activity on NET production was also tested for the most efficient proteases via the addition of the aspartic protease class inhibitor pepstatin A. No significant proteolytic effects on NET release were observed however (see below).

Our analysis of Sap influence on the netosis yield indicated that NET release is dependent on the stimulant concentration, with the highest effect evident at a protease concentration of 100 pg/ml. We observed from our analysis that Sap3 did not detectably induce NET formation, and that Sap1 and Sap7 induced netosis poorly at a concentration of 1 ng/ml, only reaching 10–15% of the positive control level. A slightly higher amount of NET production (up to 40%) was detected for Sap2, Sap5, and Sap8. However, the highest NET response levels, reaching about 60% of the positive control, were observed for Sap4 and Sap6 which are produced mainly by the hyphal form of *C. albicans*. The Sap4 concentration that was most efficient for the activation of netosis was 1 ng/ml but for Sap6 this activation occurred at a 10-fold lower protein concentration. The next most efficient Saps for NET production were Sap9 and Sap10, both of which are surface-anchored enzymes. Significantly high amounts of extracellular DNA were detectable at the Sap9- or Sap10 concentrations of 100 pg/ml.

**Figure 8:**
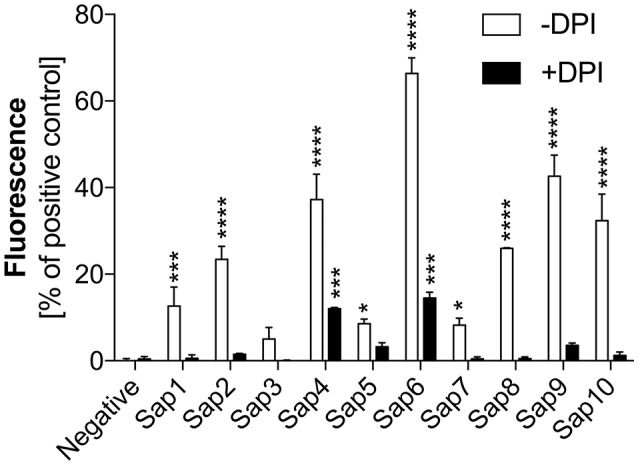
Role of NAPDH oxidase in Sap-induced netosis. Neutrophils (2.2 × 10^5^) were incubated with the indicated Saps at a concentration of 1 ng/ml for 3 h to induce netosis. Cells with active NADPH oxidase (−DPI) or with this enzyme inhibited by 5 μM DPI (+DPI) treatment were used. The amount of released DNA was determined after SytoxGreen staining. Unstimulated neutrophils served as a negative control and 25 nM PMA treatment was assumed to induce a maximal level of NET release. Data are the means ± SEM from three experiments performed in duplicate. Asterisks denote statistical significance relative to the negative control (^*^p < 0.05, ^***^p < 0.005, ^****^p < 0.001, ns—not significant).

To identify the pathway(s) involved in Sap-induced netosis, we compared NET production under protease treatment conditions with or without NADPH oxidase inhibitor (DPI) treatment. Neutrophils were stimulated with each type of Sap at a concentration of 0.1 ng/ml (Figure 8). In every case, the presence of the NADPH oxidase inhibitor led to a significant decrease in the NET response, suggesting the involvement of a ROS-dependent mechanism in Sap-triggered netosis. However, during treatment of the neutrophils with Sap4 and Sap6 in the presence of DPI, about 25–35% of the NET production level was preserved. This observation suggested that in case of Sap4 and Sap6 extracellular traps can be produced in part via a ROS-independent pathway.

### The neutrophil CD11b receptor is responsible for the triggering of netosis by Saps

**Figure 9:**
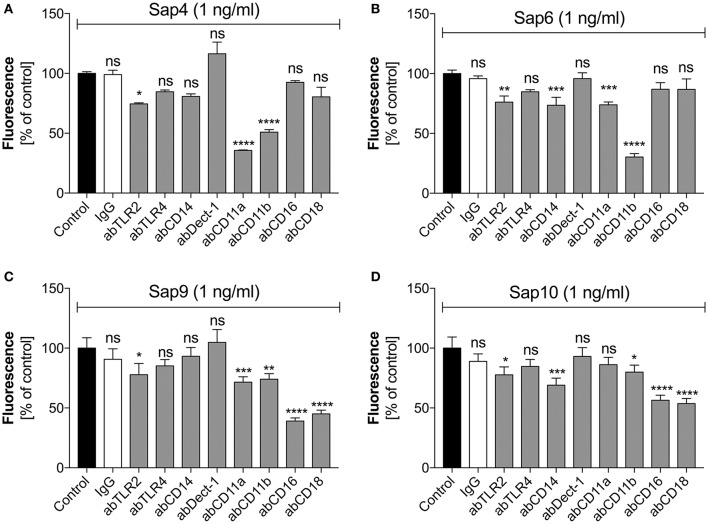
Participation of selected neutrophil receptors in Sap-triggered netosis. Neutrophils (2.2 × 10^5^) were preincubated with antibodies (**ab**, 1 μg/ml) against the indicated neutrophil receptors and netosis was then triggered for 3 h by Sap4 **(A)**, Sap6 **(B)**, Sap9 **(C)**, and Sap10 **(D)** at an enzyme concentration of 1 ng/ml. IgG antibody was used as an isotype control. The results shown are derived from three independent experiments, performed in duplicate. Data are the means ± SEM, relative to the untreated control sample. Statistical significance is denoted by asterisks (^*^p < 0.05, ^**^p < 0.01, ^***^p < 0.005, ^****^p < 0.001, ns—not significant).

For Saps with the highest NET release potency, we performed a detailed analysis of the responsible neutrophil receptors. We blocked selected receptors with specific antibodies prior to neutrophil treatment with a 1 ng/ml concentration of Sap4, Sap6, Sap9, or Sap10 (Figure 9). IgG served as a negative control. The Sap4 and Sap6 proteases, secreted mainly by the filamentous form of *C. albicans*, appeared to activate netosis via the CD11b (Mac-1) receptor, as the antibody block of CD11b led to a significant reduction in the extracellular DNA level, to about 50 or 25% of the control levels, respectively. The CD11a receptor could also be involved in this process, particularly for Sap4, as a NET response reduction of more than 50% was observed during antibody treatment for this molecule. TLR2 seemed also to play a role in the activation of netosis by Sap4 and Sap6, but at a lower level of potency. Additionally, CD14 may be involved in the triggering of netosis by Sap6.

The cell wall-anchored proteases Sap9 and Sap10 activated netosis mainly via the CD16 and CD18 receptors; a block of these two receptors led to a 50% NET response reduction. Sap9 was also found to be recognized by the CD11a and CD11b receptors; about 25% less extracellular DNA was produced by neutrophils treated with the respective antibodies against these receptors. In the case of Sap10, TLR2 and CD11b together with CD14 showed involvement in the NET response at a lower level.

**Figure 10:**
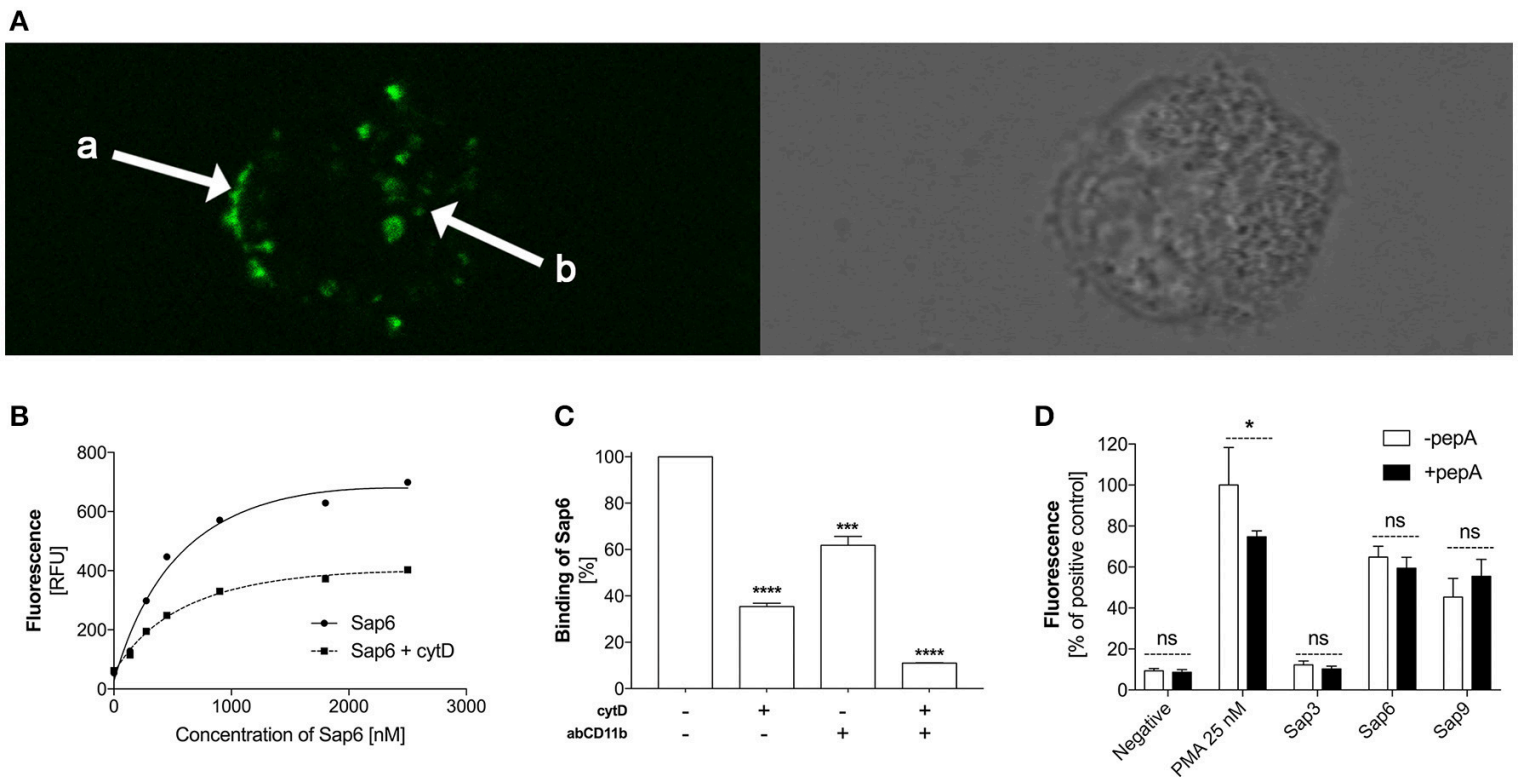
Binding of Sap6 to neutrophils. **(A)** Neutrophils (2.2 × 10^5^) were incubated for 1 h with FITC-labeled Sap6 and imaged using fluorescence confocal microscopy. Arrows indicate the localization of the protein on the surface (a) and inside the intracellular compartments (b) of the neutrophils. The left panel represents the fluorescence visualization of Saps that were in contact with the neutrophils. The right panel shows the same cell in transmitted light. Imaging was performed using an immersion lens with a magnification factor of 100x. **(B)** Interaction of neutrophils with fluorescently labeled Sap6, at concentrations of 0–2.5 μM, determined by flow cytometry. The treatments were performed for native cells **(Sap6)** and for cells with an endocytosis block using 5 μM cytochalasin D **(Sap6** + **cytD)**. Data were collected for 100,000 neutrophils for each experimental point. The results were fitted to the simplest interaction model of one class of binding sites and the estimated curve is presented. The results shown are representative of three independent experiments. **(C)** Level of fluorescently labeled Sap6 bound to neutrophils determined by microplate assay. Neutrophils in a native state **(CytD**−**, abCD11b**−**; control sample)**, with a CD11b receptor block using 1 μg/ml of antibody **(CytD**−**, abCD11b**+**)**, with an endocytosis block using 5 μM cytochalasin D **(CytD**+**, abCD11b**−**)**, and treated with a combination of these compounds **(CytD**+**, abCD11b**+**)** were used. For fitting of the interaction, the 1:1 interaction model was used. The data are the means ± SEM of two experiments performed in duplicate and are expressed as a percentage ratio relative to the control sample. **(D)** Neutrophils (2.2 × 10^5^) were incubated for 3 h without **(**−**pepA)** or with pepstatin A (10 μM) **(**+**pepA)** protease inhibitor and with Sap3, Sap6 or Sap9 at concentrations of 1 ng/ml to induce netosis. Unstimulated neutrophils served as a negative control while neutrophil stimulation with 25 nM PMA was assumed to represent the maximal level of NET release. Data are the means ± SEM from three independent experiments, performed in duplicate and are expressed as percentage ratio of the tested sample signal relative to the PMA-treated control. Asterisks denote statistical significance (^*^p < 0.05, ^***^p < 0.005, ^****^p < 0.001, ns—not significance).

We performed further analysis of the neutrophil receptor CD11b, that mainly mediates NET production under fungal protease treatment conditions, because of its interaction with Sap6, the most efficient protease in terms of netosis. Neutrophils that had settled on the plate or were suspended in the RPMI medium were exposed to fluorescein-labeled Sap6 at increasing protease concentrations for 1 h (Figure 10). Microscopic analysis of these interactions indicated that Sap6 was mainly localized on the cell surface but was also partially internalized (Figure 10A). Blocking CD11b with a specific antibody led to a significant decrease in the number of Sap6 molecules associated with neutrophils (Figure 10B). Inhibition of the endocytic activity of these cells by treatment with cytochalasin D resulted in a 45–60% lowering of the interaction with Sap6. Blocking of neutrophil binding and protease internalization completely prevented any Sap6 interaction with neutrophils and further revealed CD11b as a critical factor in this interaction (Figure 10C). Moreover, these Sap6-neutrophil interactions were not influenced by Sap6 proteolytic activity because treatment with a protease inhibitor—pepstatin A (Figure 10D)—did not change the results.

### Identification of pathways involved in Sap6-activated netosis

**Figure 11:**
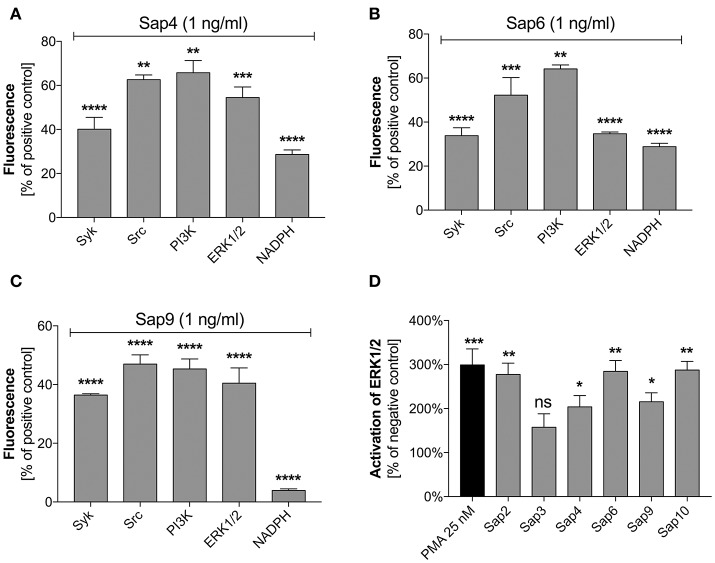
Role of selected signal mediators in Sap-triggered netosis. **(A–C)** Neutrophils (2.2 × 10^5^) were preincubated with inhibitors of the indicated signaling mediators: Syk—30 μM piceatannol, Src—10 μM PP2, PI3K—25 μM LY29004, ERK—10 μM U0126, NADPH oxidase—5 μM DPI. These cells were then incubated with Sap4 **(A)**, Sap6 **(B)**, or Sap9 **(C)** at concentrations of 1 ng/ml for 3 h to induce netosis. Neutrophils not treated with inhibitors but stimulated with Saps served as controls. The data are presented as means ± SEM from three independent experiments (in duplicate) and are expressed as a percentage ratio relative to the PMA-induced control. **(D)** Lysates were prepared for neutrophils treated with selected Saps at concentration of 1 mg/ml. The levels of phosphorylated and total ERK1/2 were then determined in these cells using an ELISA method. Unstimulated cells were used as a negative control. Data are the ratios of the phosphorylated kinase to total kinase. Data are presented as mean ± SEM of three independent experiments. Asterisks denote statistical significance relative to the negative control (^*^p < 0.05, ^**^p < 0.01, ^***^p < 0.005, ^****^p < 0.001, ns—not significant).

Specific inhibitors of selected mediators involved in the netosis pathway were used to examine the participation in NET release during contact with the most potent Sap activator, i.e., Sap6. The possible toxicity of each inhibitor toward neutrophils was excluded using the test proposed by Behnen et al. (2014). We identified Src and Syk as the main kinases involved in the induction of netosis during Sap treatment (Figures 11A–C). These mediators cooperate with selected surface receptors mentioned above, such as Dectin-1, CD16, and CD11b (Lowell, 2011; Thomas and Schroder, 2013; Behnen et al., 2014). Inhibition of Src and Syk reduced the amount of released DNA in response to exposure to different Saps by about 50%. The role of Src was found to be more important for neutrophils treated with Sap9 as compared to Sap4 or Sap6. This is probably because Sap4 and Sap6 trigger mixed netosis pathways. We speculated in this regard that the activation of Syk could transfer the signal downstream to PI3K or ERK1/2.

PI3K participates in the regulation of netosis via NF-κB (DeSouza-Vieira et al., 2016). In our current experiments, PI3K inhibition led to a decrease in NET production in response to each of the tested Saps. However, PI3K seemed to be most important for the induction of netosis by Sap9. Similarly, the inhibition of ERK1/2 kinase, which is involved in NET formation connected with ROS induction (Aleman et al., 2016), resulted in the reduced release of DNA. ERK1/2 activation was additionally examined using an ELISA test for phosphorylation of this kinase during neutrophil treatment with Saps (Figure 11D). Sap6 and Sap10 caused a three-fold higher activation of ERK1/2 compared to the unstimulated neutrophils. Lower responses were detected for neutrophils incubated with Sap4 and Sap9, thus correlating with the lower ability of these aspartic proteases to induce netosis.

## Discussion

Neutrophils that encounter the pathogenic forms of *C. albicans* use many different receptors to sense compounds present at the fungal cell surface, including the main polysaccharide components of the cell wall (glucans and mannans), adhesive surface-bound proteins and Saps. During candidal infection, the structure and properties of the cell surface of this pathogen constantly change, as exemplified by recent finding that the amount and type of cell wall-forming polysaccharides can be dynamically adjusted to make the fungal cells less recognizable by the host immune cells (Hopke et al., 2016). Accordingly, neutrophils use multiple mechanisms to cope with the pathogen attack, including phagocytosis, degranulation, and the most recently described formation of extracellular web-like structures composed of chromatin DNA and granular proteins, i.e., NETs.

In our present study, we have for the first time characterized and compared the efficiency of major classes of compounds, representing the main virulence factors of *C. albicans* that are either embedded in the fugal cell wall or secreted and thus present near the cell surface, that can stimulate neutrophils to release NETs. We propose that NET release (netosis) correlates with the ability of neutrophils to recognize these particles at the moment of infection, and involves the activation of different signaling pathways within neutrophils.

Although, several hypotheses on the causes of NET stimulation during contact with whole *C. albicans* cells have been proposed previously (Urban et al., 2006; Branzk et al., 2014; Johnson et al., 2016), our current results suggest that the actual number of pathogenic cells is the main regulating factor in the triggering of netosis. As the amount and type of *C. albicans* cells reflect the progress of infection, it seems that netosis is activated at the early stage of host infection, corresponding to an MOI of 1:1 at which stage phagocytosis cannot efficiently reduce the pathogen spread and block the development of infection. On the other hand, we found from our current experiments that an excessive number of pathogenic cells can lead to a decrease in NET formation. This observation is consistent with a previous finding that only large sized pathogen cells can trigger netosis (Branzk et al., 2014). We conclude therefore that the size as well as the number of pathogenic cells, correlating with the stage of infection, can determine the choice of defense mechanism employed by the host against *C. albicans*. Our observations are also consistent with the results obtained by Johnson et al. (2016) who reported that neutrophils showed a lowered NET release in response to the biofilm formed by *C. albicans* when the pathogenic surface in local contact with neutrophil cells was high. This idea is also supported by previous observations of lower efficiency in ROS production during pathogen contact with neutrophils. The coincubation of neutrophils with pathogenic cells at ratio of 1:10 was reported to completely block ROS generation by neutrophils (Wellington et al., 2009). Also, Johnson et al. (2016) showed that neutrophil contact with *C. albicans* biofilm significantly reduced ROS production by these phagocytes. As ROS are necessary for the main pathway of NET formation, a blockade of ROS release by a large number of *C. albicans* cells could be the reason why this causes netosis inhibition.

As the cell wall of *C. albicans* is a thick polysaccharide network composed of a deep layer of chitin, a middle layer of β-glucans and an external cover comprising mannans, and containing numerous proteins throughout (Chaffin et al., 1998; Karkowska-Kuleta and Kozik, 2015), a crucial question that arose was the identity of the major cell wall components that are most important for the induction of netosis. Among the aforementioned compounds, β-glucans are the most recognizable by immune cells that induce ROS production and phagocytosis (Figueiredo et al., 2011). The amount of glucans exposed on the surface of *C. albicans* cells has been proven to change during infection whereupon the hyphal form of the pathogen exhibits a lower production of these molecules (Gantner et al., 2003). Moreover, the layer of mannans that covers the deeper layer of glucans prevents recognition of the latter molecules, and results in a decreased immune cell response (Davis et al., 2014).

Although, β-glucans are a well-known stimulus of netosis, the signaling pathways involved in this process are still under discussion (Byrd et al., 2013, 2016; Nanì et al., 2015; Johnson et al., 2016). In our present analysis, we observed that glucans activated netosis in a dose-dependent manner, mainly via a ROS-dependent signaling pathway. These results are similar to those obtained by Nanì et al. (2015). On the other hand, Byrd et al. (2013, 2016) reported that glucan-induced netosis is ROS-independent and requires a fibronectin base. In contrast to this however, Nanì et al. (2015) recently proposed that fibronectin is not required at the contact surface. Although, our current study findings indicate that NET release upon neutrophil treatment with glucans is dependent on the efficient activity of NADPH oxidase, the possible role also of a ROS-independent mechanism cannot be excluded by our results. Some of the inconsistencies between our present results and those of Nanì et al. or Byrd et al. could be due to the different types of β-glucans used in each study. An advantage of our current study was the use of β-glucans isolated from *C. albicans* cells, in contrast to soluble β-glucans purified from *Saccharomyces cerevisiae* that were used by other authors (Byrd et al., 2013, 2016; Nanì et al., 2015). We found that netosis triggered by glucans involved two groups of receptors: Dectin-1 (a C-type lectin receptor) and CR3 receptor (Mac-1; CD11b/CD18). Dectin-1 is known to be one of the major receptors involved in β-glucan recognition (Brown and Gordon, 2001; Brown et al., 2002, 2003; Li X. et al., 2011) with ROS induction and initiation of phagocytosis as the responses. Mac-1 was also shown to be an important player in β-glucan recognition by neutrophils (van Bruggen et al., 2009), which is followed by phagocytosis. On the other hand, Byrd et al. (Byrd et al., 2013, 2016) reported that ROS-independent netosis was activated by the CR3 receptor, but not by Dectin-1. Nanì et al. (2015) in turn suggested that NETs released via a ROS-dependent pathway engaged the Dectin-1 receptor. They also reported that inhibition of NADPH oxidase did not completely inhibit NET release following contact with β-glucans. Moreover, another group described the activation of the Mac-1 receptor by glucans as indirect and to be the result of Dectin-1 activation (Li X. et al., 2011). All of these previous insights support our present conclusion that β-glucans exert a wide influence during netosis and, in combination with the action of other compounds presented on the cell surface, could be a central process by which neutrophils ensure an adequate host immune response to *C. albicans* infection, especially when the mechanism of ROS production is inhibited by this pathogen (Wellington et al., 2009).

Mannans, which are other polysaccharides that comprise the candidal cell wall, are also recognized by neutrophils (Hall and Gow, 2013; Netea et al., 2015). This is important for the host because these polysaccharides often cover the layer of β-glucans on the *C. albicans* cell surface, rendering it less visible to the host immune system (Bain et al., 2014; Davis et al., 2014). Our present results indicated that neutrophils can detect mannan particles and initiate a NET response to this in a dose-dependent manner. Because the mannan shield appears, in particular, during the transition from the unicellular to hyphal form of *C. albicans* (Gantner et al., 2005) we hypothesize that the amount of mannans on the cell surface may be an indicator of infection progression, pointing to the need for a diverse set of mechanisms to be utilized by the host response system. This interpretation was further supported by our observation that an excess concentration of mannans or increasing MOI of contacting fungal cells did not produce a NET release. Our results are also consistent with the findings of Johnson et al. who reported that a reduction of *C. albicans* mannosylation during biofilm formation led to an increased NET formation upon contact between phagocytes and pathogenic cells (Johnson et al., 2016). Moreover, mannan-triggered netosis seems to be independent of ROS generation or, at least, to utilize both mechanisms. This possibility explains why many types of receptors tested in our current analysis seem to be involved in the neutrophil response to mannans. The first group of receptors we tested included the TLR2 and TLR4 neutrophil receptors whose connection with NET release was demonstrated previously (Tada et al., 2002; Netea et al., 2006; Figueiredo et al., 2011). Together with the TLRs, the CD14 receptor also takes part in the identification of fungal compounds and the filamentous form of fungi (Figueiredo et al., 2011). This receptor contributes also to the recognition of mannans (Tada et al., 2002) and, as we documented in our present experiments, is involved in netosis activation. We further described the roles of Dectin-1 and CD11b in NET formation by mannans. Notably, to our knowledge, there has been no information provided previously in the literature about the role of these receptors in mannan recognition.

The *C. albicans* cell wall also contains proteinaceous compounds, the most important group of which comprises the adhesins that are capable of binding to many host proteins and/or become involved directly in adhesion processes. A representative of this group evaluated in our current study was Als3, a prominent adhesin often overexpressed and detected in candidal infections (Liu and Filler, 2011). However, we did not find any evidence for its involvement in netosis. In contrast, our current results showed that neutrophils released NETs upon contact with enolase—the cytosolic enzyme involved in the glycolysis pathway and also known to be exposed on the cell surface of numerous pathogens where it can “moonlight” as an adhesin that is important for pathogen cell adherence to the host tissues (Karkowska-Kuleta and Kozik, 2014). Similar NET-inducing effects were observed previously for α-enolase from *Streptococcus pneumoniae* (Mori et al., 2012). Nevertheless, we found that *C. albicans* enolase seemed be a far less potent activator of netosis as compared to the polysaccharides mentioned above, and to only do so within a relatively narrow concentration range. However, a role of enolase in netosis cannot be excluded, particularly during the formation of biofilms in which this protein has been found to cover *C. albicans* cells.

Another important group of *C. albicans* virulence factors is the family of 10 Saps. These enzymes are expressed at various combinations and at different places and stages of infection (Naglik et al., 2008; Pietrella et al., 2010) and have been proven to support the invasion of *C. albicans* cells into host tissues (Staniszewska et al., 2012). All of the *C. albicans* Saps are released as soluble forms into the surrounding medium except for Sap9 and Sap10 which are covalently attached to the fungal cell wall. Neutrophils can identify Saps as chemotactic agents that induce further inflammation (Naglik et al., 2003a; Pietrella et al., 2013; Pericolini et al., 2015; Gabrielli et al., 2016). The most convincing results in our current analysis that indicated the significance of Saps for netosis activation came from the comparison of NET release during the contact of neutrophils with wild and Sap-knockout mutant strains of *C. albicans*. In this analysis, the elimination of dedicated proteases resulted in a changed netosis response in correlation with an increased MOI of contacting cells (Figure 6). These observations were consistent with the results from experiments performed with purified enzymes. For all 10 Saps analyzed in our current experiments, we compared the effect of NET release at different enzyme concentrations. However, it must be noted that a knockout of one enzyme from the Sap family can be compensated for by the increased expression of another Sap to rescue virulence deficiencies (Naglik et al., 2003a, 2004, 2008; Albrecht et al., 2006). The strongest response was previously observed for Sap4 and Sap6, which are dominant for the filamentous form of *C. albicans* (Hube et al., 1994; Naglik et al., 2003a) which is more virulent than the unicellular yeast-like form and induces a stronger NET response upon contact with neutrophils. The importance of these Saps in *C. albicans* infection is not only due to their proteolytic properties, but also to their role in fungal cell integrity and adherence (Naglik et al., 2003a; Buu and Chen, 2013; Kumar et al., 2015).

The markedly lower response of neutrophils to Sap1-Sap3 treatments corresponded to their predominant expression in the yeast-like form of *C. albicans* (Naglik, 2014) which is a less active inducer of netosis. The lowest potency found for Sap7 in terms of NET induction could be explained by its lowest sequence similarity to other members of Sap family and its different structural properties (Aoki et al., 2012). Moreover, the *SAP7* gene is often not expressed during infection and its role as a virulence factor has not been well established (Naglik et al., 2003b, 2008; Naglik, 2014). The response of neutrophils to contact with Sap8 and their recognition of the mutant *C. albicans* strain containing a Sap8 deletion suggested possible neutrophil activation by this Sap to form NETs but the mechanisms by which Sap8 may contribute to human infection in general remain unclear (Carvalho-Pereira et al., 2015).

Sap9 and Sap10 are closely related and are linked via a GPI anchor to the *Candida* cell wall (Monod et al., 1998; Schild et al., 2011). These enzymes represent the second group of proteases that strongly support netosis. This newly presented role of these Saps from our current analysis broadens our understanding of their ability to participate in *C. albicans* infection, and supplements their previously known roles in cell growth, cell surface integrity and adhesion (Naglik et al., 2008; Nailis et al., 2010; Schild et al., 2011), and biofilm formation (Dutton et al., 2016).

Our attempts to determine the mechanisms involved in the activation of netosis during neutrophil contact with the most effective netosis-inducing Saps revealed some dual actions. The activity of NADPH oxidase and, hence, ROS production appeared to be important for Sap-induced netosis, mainly upon the contact of neutrophils with Sap1, Sap2, Sap8, Sap9, and Sap10. On the other hand, neutrophils treated with an NADPH oxidase inhibitor and with Sap4 and Sap6 still released NETs, but the amount of DNA was significantly reduced. This suggests the possibility of a selection between ROS-dependent and ROS-independent mechanisms in this case. The activation of ROS-independent processes as an alternative pathway could be a mode of host protection against immune response inactivation by ROS during fungal or bacterial infection (Wellington et al., 2009). However, the role of this dual mechanistic response during netosis triggering still remains unclear. Further analysis of involved receptors and concomitant activation of the corresponding signaling pathways could shed more light on this issue. However, it is worth noting that closely interlinked signaling events cannot be assigned to the action of a single receptor (Duggan et al., 2015).

Sap4 and Sap6, which can activate netosis by ROS-dependent and ROS-independent mechanisms, were recognized during this process mainly by CD11b (Mac-1) and CD11a (LFA-1) receptors. Moreover, Sap6 was also found to stimulate the CD14 receptor. Mac-1 participates in many different types of neutrophil responses, including phagocytosis, internalization and netosis (Whitlock et al., 2000; Behnen et al., 2014). It also causes the downstream activation of the Src/Syk kinase family (Whitlock et al., 2000; Behnen et al., 2014). In turn, the CD14 receptor can serve as a co-receptor for TLR4 (Zanoni et al., 2011) and CD16 (FcγRIIIB) (Detmers et al., 1995). As we have shown from our current findings, the activation of netosis by Sap9 and Sap10 occurs through the action of CD16 (FcγRIIIB) and CD18 receptors. The participation of CD16 in the recognition of immune complexes, as well as in the induction of netosis, has been documented previously (Aleman et al., 2016). Moreover, CD16 can cooperate with the Mac-1 receptor (Zhou and Brown, 1994) and could be an important factor in the mixed mechanism activation of netosis. Additionally, a weaker involvement of the CD11a and CD11b receptors in Sap9 recognition and the CD14 receptor in Sap 10 recognition were also observed in our experiments.

The participation of a number of cooperating receptors in the identification of the Saps leads to netosis activation through the same signal-transducers. We examined the role of four signal mediators, Src, Syk, PI3K, and ERK, in this regard. The role of Src/Syk kinases in NET formation was confirmed previously (Behnen et al., 2014; Nanì et al., 2015; Aleman et al., 2016), pointing to the Src family kinases as the key signaling mediators activated by the CD11b receptor as well as the CD16 receptor (Lowell, 2011; Behnen et al., 2014), with further activation of Syk and downstream signal transduction via the activation of PI3K (Lowell, 2011) and ERK1/2 (Whitlock et al., 2000; Behnen et al., 2014). The identity of the receptors involved in the neutrophil response to Saps suggests that the Src/Syk kinases also play an important role in netosis via activation by *C. albicans* virulence factors. Indeed, our current finding of a reduced NET response during the inhibition of Src or Syk in neutrophils treated with Saps has confirmed that hypothesis.

It must be noted that the role of PI3K in netosis is still unclear. Aleman et al. suggested previously that NET induction by CD16 did not require PI3K activation (Aleman et al., 2016) but Behnen et al. described an important role of this mediator in netosis (Behnen et al., 2014). Moreover, PI3K activation in parasite-induced NET formation was confirmed in another study (DeSouza-Vieira et al., 2016). PI3K generates phosphatidylinositol (3,4,5)-trisphosphate (PIP_3_) which regulates the transcription factor NF-kB (Ward et al., 2004), promoting proinflammatory responses and NET formation (Lapponi et al., 2013). Our current results indicate however, that PI3K inhibition led to only a partial reduction in extracellular DNA release by neutrophils in response to Saps.

Another important signal mediator, ERK1/2, has also been proposed to be involved in NET induction (Hakkim et al., 2011; Behnen et al., 2014; Aleman et al., 2016; DeSouza-Vieira et al., 2016) but its precise role in netosis is still unclear. Some studies have reported that the upstream activation of ERK1/2 is essential for ROS formation (Hakkim et al., 2011) but others have found that ERK1/2 activation was downstream-regulated by the activity of NADPH oxidase (Keshari et al., 2012). In our present models of neutrophil treatment with Saps, we confirmed the contribution of ERK1/2 to the NET release process. It is worth noting also in this regard that the activation of ERK1/2 by Syk kinase involves TLR receptor mediation, which activates ERK1/2 via the IRAK family of proteins (Futosi et al., 2013). Moreover, IRAK kinases may activate the NF-kB signaling pathway, which could be particularly important for Sap6 and Sap10. These two Saps seem to induce netosis also via the CD14 receptor, a co-receptor for the TLRs.

In our more detailed analysis of the Sap6 interaction with neutrophils, as the most potent NET-generating protease, we observed that the Mac-1 receptor could interact directly with Sap6 at the surface of the neutrophils. However, a Mac-1 block did not completely reduce this interaction, suggesting that the interplay between Sap6 and neutrophils is more complex and may involve other integrins. This finding directed our attention to the surface-exposed integrin-binding motif (RGD) that is present only in Sap 4 (a single RGD motif), Sap5 (an RGDKGD motif), and Sap6 (two sequential RGDRGD integrin-binding motives) and is involved in interactions with integrin on epithelial cells (Wu et al., 2013). No other Saps contain this motif which may partially explain the strongest and ROS-independent neutrophil response to Sap6. It is also worth noting that variations in the RGD motif affect the binding affinity to a particular integrin molecule (Scarborough et al., 1993). Hence, the divergence of this motif between Sap5 and Sap6 could underlie the differences between these proteases in triggering NET release. Moreover, our current results also strongly suggest a very tight binding between neutrophil integrin and hyphal Saps. Similar interactions were also reported previously for fungal cell aggregation and virulence (Kumar et al., 2015).

Further effects of Sap6 interaction with neutrophils, resulting in NET production, seem to be connected with its internalization, a well identified process for the activation of the inflammasome in monocytes where internalization of Sap6 via clathrin-dependent mechanism is required (Pietrella et al., 2013) and is connected with ROS production, or for the induction of epithelial cell apoptosis via a “Trojan horse” mechanism (Wu et al., 2013). The internalization of Sap6 by neutrophils may be vital for a fast response to *C. albicans* invasion, probably through a ROS-independent netosis pathway. However, this issue requires further studies to elucidate the pathways more precisely.

**Figure 12:**
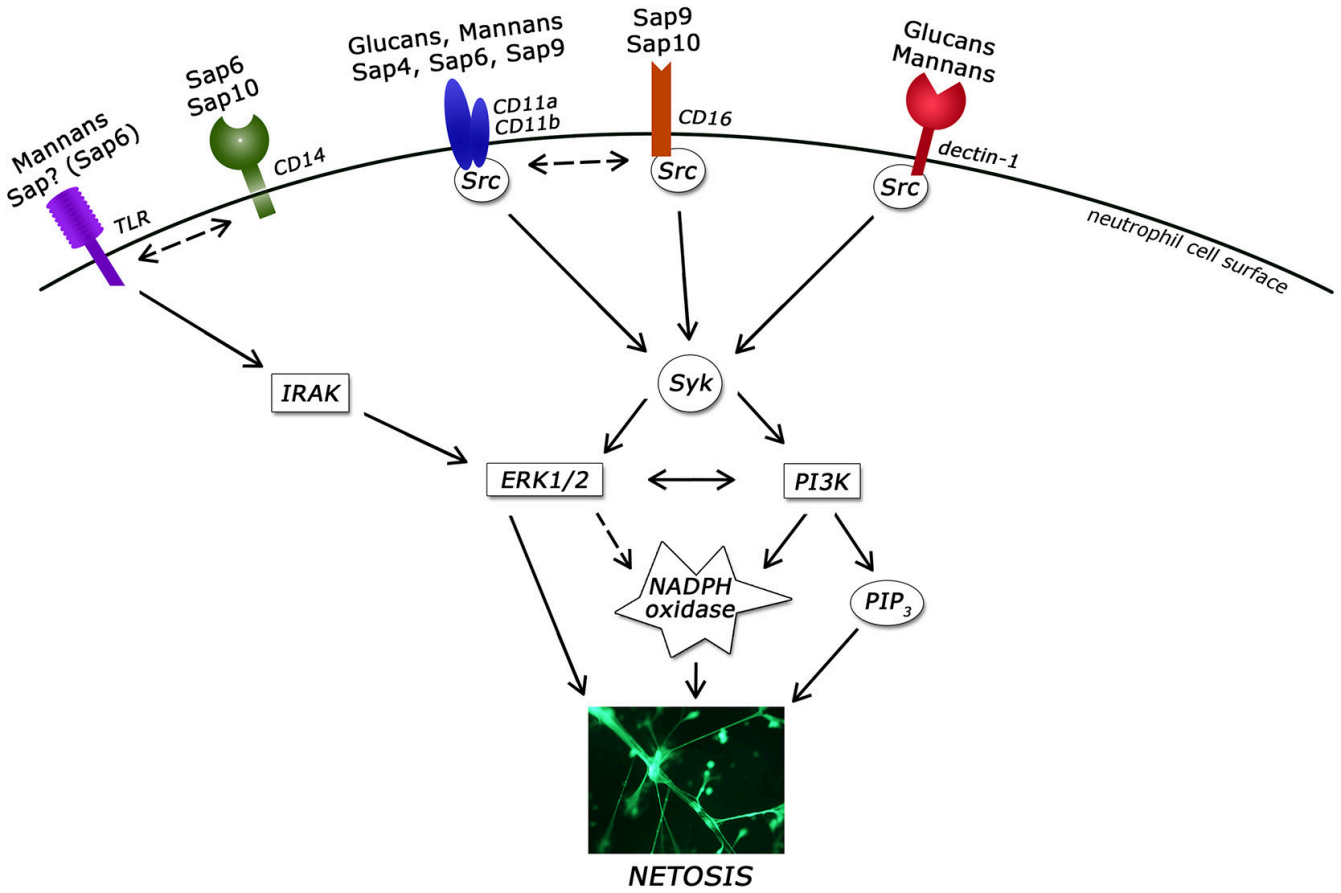
Involvement of neutrophil receptors and signaling pathways in NET activation by the main virulence factors of *Candida albicans*. ERK1/2, extracellular signal–regulated kinases; IRAK, Interleukin-1 receptor-associated kinases; Src, Src kinase family; Syk, spleen tyrosine kinase; PI3K, extracellular signal–regulated kinases; PIP_3_, phosphatidylinositol (3,4,5)-trisphosphate; PKC, protein kinase C; TLR, Toll-like receptor.

The information in this study concerning NET release following neutrophil contact with the known *C. albicans* virulence factors are summarized in Table 2 and presented in Figure 12.

**Table 2:** Summary of netosis induction by virulence factors at the cell surface of *C. albicans*.

**Component of *C. albicans***	**Induction of netosis**	**Pathway**	**Involved receptors**
β-Glucan	Yes	ROS-dependent	Dectin-1, Mac-1
Mannan	Yes	Mixed^*^	Dectin-1(?), CD14, Mac-1, TLR2, TLR4
Moonlight proteins (enolase)	Yes (poorly)	–	–
Adhesin (Als3)	No	–	–
Sap1	Yes	ROS-dependent	~
Sap2	Yes	ROS-dependent	~
Sap3	No	–	~
Sap4	Yes	Mixed^*^	CD11a, CD11b
Sap5	No (poorly)	–	~
Sap6	Yes	Mixed^*^	CD11b, CD11a, CD14, TLR2
Sap7	No (poorly)		~
Sap8	Yes	ROS-dependent	~
Sap9	Yes	ROS-dependent	CD16, CD18, CD11a, CD11b
Sap10	Yes	ROS-dependent	CD16, CD18, CD14

* *ROS-dependent and ROS-independent; ~, not analyzed*.

## Author contributions

Conceived and designed the experiments: MR and MZ. Performed the experiments: MZ, OB, JK, and KS. Preparation of plasmids: WA and MU. Analyzed the data and wrote the paper: MZ and MR. Revised it critically: AK.

### Conflict of interest statement

The authors declare that the research was conducted in the absence of any commercial or financial relationships that could be construed as a potential conflict of interest. The reviewer FA and handling Editor declared their shared affiliation.
